# Early History of Veterinary Medicine in the United States

**Published:** 1883-01

**Authors:** R. Jennings


### Art. VII.—THE EARLY HISTORY OF VETERINARY MEDICINE AND SURGERY IN THE UNITED STATES.

BY R. JENNINGS, V. S.

BY special request, I reluctantly undertake the task of writing a brief synopsis of the early efforts made to introduce a higher standard of veterinary knowledge in the United States, and as a matter of history, to place it upon record. At the same time I am compelled to admit my inability to do the subject that justice which I believe it fully merits. I was the first in the country to move in this arduous undertaking, at the time when the practice of veterinary medicine and surgery was in a low and degraded condition, confined mainly to the hands of a very illiterate and intemperate class of men, whose treatment of the sick animal was characterized by ignorance, absurdity, barbarity and superstition. In my early days I witnessed many cruelties upon horses and cattle under the treatment of the farrier, which if practised at the present time would subject the operator to criminal prosecution. Some of these were the infliction of the stifle shoe; the burning of the bars of the mouth in lampas; the pricking and docking of the tail; inserting silver coin in the atrophied shoulder; the inflation of the cellular tissue with air, and many other like devices. The necessity for a radical change, and the diffusion of correct veterinary knowledge throughout the agricultural districts was the incentive which prompted my efforts in this direction. As there were no veterinary colleges in this country at that time, and as I had not the pecuniary means necessary for a trip to Europe, in order to gain a thorough veterinary education, I entered the office of the late, T. J. Corbyn, (then the leading veterinary surgeon in the City of Philadelphia), as a student, and to assist me farther I matriculated at the Pennsylvania Medical College, Philadelphia. During the winter months of 1846 to 1850 I delivered a course of veterinary lectures each year, to small classes of students from the several medical colleges in the city, whose future field of usefulness were the agricultural districts. About the year 1850 I conceived the idea of organizing a veterinary college, to be located in the City of Philadelphia, and for that purpose made known my plans to my medical preceptor, Prof. James Bryan, formerly of the Geneva Medical College, New York, and through him to Prof. Wm. Gibson, of the Pennsylvania Medical University—the oldest Medical College in the United States. Through their influence the following named citizens, Geo. Cadwallader, John Philips, M.D., Alfred L. Elwyn, M.D., Fred'k Watts, George M. Keim, Hon. George W. Woodward, Sketchley Morton, Alonzo Potter, M.D., and L. L. Ward, readily subscribed to the contemplated veterinary college, and made up a list of $40,000. An application was now made to the State Legislature, for a charter which was granted on the 15th day of April, 1852.

This, the pioneer Veterinary College of America, was destined to meet with many disappointments, and much opposition from those who should have extended a helping hand. The graduates of European schools, with few exceptions, gave the movement the cold shoulder, regarding the effort as premature, resorting to various, and sometimes disreputable means to discourage those most active in its success. The only recognized members of the profession in the city, who were co-workers in the cause, using their influence and their means, in behalf of the infant institution, were, Thos. J. Corbyn, W. W. Fraley, John Scott, and the writer, who constituted the faculty. An anouncement was issued for a regular course of veterinary lectures, upon the several branches pertaining to a thorough knowledge of veterinary medicine and surgery, to commence the first Monday in November, 1853, continuing daily for sixteen consecutive weeks, which announcements were freely distributed, bringing letters of inquiry in return, but no students; young men of education and respectability, would not engage in a profession of so low a standing. In that day the title of veterinary surgeon, was rarely heard, and but few persons in this country, understood its meaning. The members of the profession were known only by the appellation of "Farrier," "Horse or Cow Doctor." Johnsons Dictionary defines *Farrier* thus; "To practice physick or chirurgery on horses." Walker—"A shoer of horses, one who professes the medicine of horses." Webster—"A smith who shoes horses, a veterinary surgeon." Failing to secure a class, T. J. Corbyn, W. W. Fraley, and John Scott tendered their resignations as professors of the college, leaving the writer alone to fight its battles. Nothing daunted, young and ambitious, I sought to harmonize the discordant spirits by bringing the members of the profession in friendly counsel, and urging them to unity of action. A meeting of the veterinary surgeons in the city was called and the subject discussed in all its bearings. After several meetings were held a permanent organization was effected the 7th day of May, 1854, under the title of the American Veterinary Association, and the following officers were elected to serve one year. President, T. J. Corbyn, V. S.; Vice-Presidents, James Bryan, M.D.; and W. W. Fraley, V. S., Secretary; M. Roberts, V. S., Corresponding Secretary; John Scott, V. S., Treasurer; R. Jennings, V. S., Librarian; A. Tegtmeier, V. S., Counsellor; Marcellus Munday, Esq. *Senior members,* T. J. Corbyn, R. Evans, W. W. Fraley, R. Jennings, M. Roberts, J. Scott, and Aug. Tegtmier.

*Patrons*—James Bryan, M.D., Wm. Gibson, M.D.

ARTICLE II. OF THE BY-LAWS, SAYS:

"Its objects shall be the cultivation of fraternal feelings among veterinary practitioners; the elevation of the veterinary art (meaning thereby the treatment of diseases occuring in our domesticated animals), to an equal rank with other scientific branches of medicine, the mutual improvement of its members, by the presentation of such cases of disease, together with their treatment and termination, that may come under the notice of any gentleman belonging to the association, which may be deemed of sufficient interest to bring before the Society; the establishment of a Museum of Anatomical and Pathological Specimens; and the formation of a Library, consisting of such works as are necessary to elucidate and impart information on Veterinary Science; and in general, the defence of the rights, and privileges and immunities of the veterinary practitioners in the United States."

At its Exhibition held in September, 1854, in the City of Philadelphia, the Pennsylvania State Agricultural Society recognized this association, by awarding it the highest premium, a silver medal, bearing the following inscription. "Awarded to the American Veterinary Association for Pathological Specimens, at the Exhibition of 1854." This collection was prepared for the Museum of the Veterinary College of Philadelphia. In December of the same year (1854) Dr. Geo. W. Bowler arrived in Philadelphia, from England. Making his acquaintance I found in Dr. Bowler an able and energetic co-worker. The Trustees of the college pleased with this new acquisition, at once accepted him as a member of the new faculty, consisting of only Dr. Bowler and myself.

It now being evident that a full faculty could not be obtained in Philadelphia, with the opening of the spring of 1855, we started on a mission in the interest of the new school, making a tour through the states of New York, Pennsylvania, and Ohio, meeting many sympathizing friends in our travels, but no material encouragement to the new venture. Failing in our mission, Dr. Bowler, settled down in Cincinnati, where he still remains. At the same time I accepted the position of Veterinary Lecturer in the Ohio State Agricultural College; then located on the Heights, Ohio City, now West Cleveland, retaining that position until the suspension of the college in the year 1857. I then returned to Philadelphia, and again renewed my efforts in behalf of the veterinary college. Soon after my return, I presented the Trustees the following names, (subject to their approval) as constituting the Faculty of the Veterinary College of Philadelphia.

W. W. Fraley, V. S. *Prof. of Materia Medica and Therapeutics.*
T. J. Corbyn, V. S., *Prof. of Pathology and Surgery.*
Aug. Tegtmier, V. S., *Prof. of Chemistry and Pharmacy.*
R. Jennings, V.S., *Prof. of Comparative Anatomy and Physiology.*

These names meeting the approval of the Trustees a suitable building, for temporary use, was secured and fitted up to meet the wants of the Institution. Located at the corner of Sixth and Master Streets.

A dissecting room was built upon the Knackers grounds, where material was always at hand. The Trustees now called a meeting, for the purpose of discussing the propriety of securing a lot and erecting a building adapted to the wants of such an institution, and negotiations were entered into for a site on North Broad Street. John Notman, Architect, was employed to draft a plan for the contemplated building, which was in due time submitted to the Directors and Faculty, and was accepted. The plan consisted of two lecture rooms, a dissecting room, museum, laboratory, hospital accommodation for thirty patients including twelve box stalls, shoeing forge and operating room, with an operating table so constructed as to secure the patient in a standing position, or by means of a crank to lay him upon the side at a proper elevation. Previously to the purchase of the lot however, dissensions arose among the members of the profession, which threw a gloom over the future prospects of the college. The Directors and Incorporators becoming discouraged, abandoned the projected building. Had harmony prevailed, Philadelphia to day could boast of her Veterinary College.

The following extract from "*Porter's Spirit of the Times,*" June 15th, 1858, will be read with interest by friends of the cause.

"PROGRESS OF VETERINARY SCIENCE IN PHILADELPHIA."

---

*Dear Spirit*—We beg leave to call the attention of your readers to the efforts now being made in Philadelphia, to establish a Veterinary College. After a long struggle on the part of the veterinary talent hereabouts, for the attainment of this object, the road is finally open to complete success; and it is probable that, in a few months, the number of pupils, the facilities for instruction and the necessities arising therefrom, will compel the originators of the plan to announce a faculty, and in other respects assume for the enterprise the character of a regularly chartered and appointed College. A few gentlemen met in the vicinity of the "Knackers." on the 8th instant. at Myer's Place, situated in the northern part of this city. The call was a very limited one, made by a few Veterinary Surgeons, who have been contributing their weight of talent and mite of labor to the grand object in view. Consequently, the gathering was small and "sweetly select,"—confined principally to the actual and interested members of the American Veterinary Association, which by the way, has its origin also in Philadelphia. Your reporter, as an invited guest, had the opportunity of inspecting the premises selected by Drs. Scott, Fraley, and other active movers in the enterprise, for their present labors. They have a dissecting-room and museum, which are apartments contiguous to a spacious barn and stabling used as an infirmary. The dissecting-room is complete in all its appointments, and is a busy and profitable resort for students in anatomy. The great mortality in horse-flesh would constantly furnish enough subjects for dissection to employ the attention of any number of pupils. With such opportunities and facilities for observation and collection, the natural consequence is the existence of a museum, already large and valuable. There is not such a collection in the United States: and, as the property of the American Veterinary Association, it has won every praise for its contributors, and a prize at a late Fair of the State Agricultural Society of Pennsylvania. As a mere museum of curiosities, it is sufficiently interesting; as a collection of anatomical and pathological specimens in hippology, it is sufficiently valuable; but as the nucleus for the accumulation of facts in the comparatively little-understood subjects of the hygiene, the diseases, the accidents, and their management in the horse, it is grand. It is upon such facts that inductive logic bases its theories, which, when modified by deductive experiments become the key to the mysteries of treatment, and the *signa crucis* to the fount of the laws of all natural phenomena.

While inspecting the osteological portion of the museum, we were struck by the grand and extensive field here presented for the study of human anatomy and pathology. An address was delivered by Dr. Jas. Bryan; this was in substance, the same address, or "Plea for the Establishment of Veterinary Colleges in the United States," delivered on a former occasion before the State Agricultural Society of Pennsylvania. The history of the American Veterinary Association was briefly told, and the importance of the establishment of colleges duly set forth. Such is the substance of Dr. Bryan's very pleasing address. He is an eminent physician of Philadelphia, and in no way connected with the Veterinary Association, except as patron; but his whole heart and education are devoted to the advancement of science in all its departments. We have now done a portion of our duty in making this report. We invite the close attention and aid of the veterinary talent of your city to the subject. Philadelphia has thus far taken the lead; but, without doubt, you present a wider field and more extensive material to insure success. Stir up your medical men, as has been done here. Induce your Brians, your Woodwards, your Ingersols, your Cadwalladers, your Florences, your Potters, for you have the noble counterparts of these Philadelphia names in Medicine, in Law, in Divinity, and in Political Economy—induce them to put their shoulders to the wheel and actively support the effort. We predict that a time is soon coming, when the social and professional position of veterinary practitioners, and the aims and character of Veterinary Science in general, will rapidly attain an elevation worthy the responsibility and importance of the noble calling. The formation of colleges, by virtue of their legal appointments, will "secure the defence of the rights, priveleges, and immunities" of their Alumni, "and of the profession at large."

The early history of Veterinary Medicine and Surgery does not differ much from that of other useful sciences which have revolutionized the world. Its beginnings were small and seemingly unimportant.

The Boston Veterinary Institute was chartered in the year 1855. Its rise and progress can best be portrayed by a Thayre, a Saunders, or a Stickney. In the same year the Veterinary College Institute of New York, was chartered under the direction of Capt. John C. Ralston, M.D., C. V. S. formerly a veterinary surgeon in the British Army. An imposing building was erected on West Twenty-third Street, at a cost of $40,000 with hospital accomodations for fifty patients. After two years struggle the object for which it was erected was abandoned. The New York College of Veterinary Surgeons, was chartered in the year 1857, which charter was amended in 1862. A building on Lexington Avenue was secured and nicely fitted up for the purpose for which it was intended. But for some reason of which we are not informed it did not prove a success. But to return to the Veterinary College of Philadelphia. Failing to secure a class of students for its proposed sessions, of 1857-'58 and also for 1858-'59 it renewed its efforts for the session of 1859-'60; which efforts were awarded by the application of two students, viz.—Jacob Dilts, of Lambertsville, New Jersey, a graduate of the Boston Veterinary Institute, and W. Wisdom, of Wilmington, Delaware, who had been practising Veterinary Medicine and Surgery for nearly thirty years, a portion of which time was in the City of New York. With these two students the first session of the Veterinary College of Philadelphia commenced. Unfortunately for the new institution the course of lectures had scarcely begun, when, from some cause not explained, W. W. Fraley, T. J. Corbyn, and Aug. Tegtmier tendered their resignation as professors in the College. By direction of the Trustees, I at once wrote for Dr. G. W. Bowler, who promptly responded to the call. The expenses falling heavily upon the purses of the trustees and faculty, who received no pecuniary recompense for the services, an appeal was made to the Philadelphia Society for the promotion of agriculture, for assistance, which appeal was referred to the executive committee, who, at the next meeting of the Society, made the following report, relative to the application—that they had visited the library and museum of the veterinary college, and were surprised to find it so valuable and interesting. They were so favorably impressed that they ceased to doubt the propriety of the application; and therefore offered a resolution: That the use of the rooms of the Agricultural Society, be granted to the Veterinary College of Philadelphia, for holding their lectures during the winter session. Dr. A. L. Elwin, seconded the resolution and spoke earnestly in favor of inviting the faculty of the veterinary college, to deliver a course of lectures upon hippology, under the auspices of the society, which was agreed to unanimously. The College continued to hold its sessions in the Agricultural Hall, until its suspension in 1866.

(*To be contiuued.*)

